# Anxiety, Depression, and Stress on School-Aged Children and Adolescents in 2021: An Urgent Need for Comprehensive Intervention and Support

**DOI:** 10.1192/j.eurpsy.2024.925

**Published:** 2024-08-27

**Authors:** A. Romero, M. Petro, E. P. Ruiz

**Affiliations:** ^1^CORDOBA, Universidad de Cordoba; ^2^CORDOBA, GEORGES NOBLE SCHOOL; ^3^CORDOBA, Universidad Pontificia Bolivariana, MONTERIA, Colombia

## Abstract

**Introduction:**

In the year 2021, there was a notable increase in behaviors associated with anxiety, depression, and stress among school-aged children and adolescents, possibly attributed to the pervasive effects of social isolation and confinement measures.

**Objectives:**

This study conducted a thorough analysis of cases involving students aged 11 to 17 years who exhibited risk factors, anxious and depressive symptoms, and mood disturbances.

**Methods:**

This study focused on students aged 11 to 17 years and employed a comprehensive approach to assess the impact of anxiety, depression, and stress. Cases were meticulously analyzed, and key categories were established to characterize the multifaceted challenges faced by the students. These categories included the availability of family support, utilization of psychopharmacological treatment, engagement in psychological therapies, participation in psychopedagogical interventions, and patterns of school absenteeism.

**Results:**

The analysis revealed a concerning prevalence of anxiety, depression, and stress-related symptoms among the student population. Many students exhibited risk factors that warranted immediate attention, including social isolation, disrupted routines, and uncertainty about the future. Furthermore, a significant portion of students displayed anxious and depressive symptoms, often leading to altered mood and behavioral challenges. In the context of family support, it was apparent that students with robust familial backing tended to cope more effectively with the psychological strain induced by the pandemic. However, a noteworthy number of students lacked adequate family support systems, exacerbating their mental health struggles. Students in need of such interventions benefited significantly from their implementation, demonstrating improved emotional well-being and a reduction in symptom severity. Nonetheless, the accessibility of these services remained a concern, with disparities in access evident among different demographic groups. Psychopedagogical interventions played a pivotal role in addressing issues related to school absenteeism and facilitating a smoother transition to remote learning. Students who engaged in these interventions showed positive progress in terms of school attendance and academic performance.

**Conclusions:**

The findings of this study underscore the urgency of a holistic approach to addressing anxiety, depression, and stress in school-aged children and adolescents. It is imperative that consultations with child and adolescent psychiatry specialists be conducted promptly and in a manner that considers the unique contextual factors influencing each student’s mental health. Moreover, efforts should be directed toward enhancing family support, expanding access to psychopharmacological treatment and psychological therapies, and promoting the implementation of psychopedagogical interventions.

**Disclosure of Interest:**

None Declared

